# Efficacy of closed reduction for developmental dysplasia of the hip: midterm outcomes and risk factors associated with treatment failure and avascular necrosis

**DOI:** 10.1186/s13018-020-02098-3

**Published:** 2020-12-02

**Authors:** Ge Zhang, Ming Li, Xiangyang Qu, Yujiang Cao, Xing Liu, Cong Luo, Yuan Zhang

**Affiliations:** Department of Orthopaedics; Ministry of Education Key Laboratory of Child Development and Disorders; National Clinical Research Center for Child Health and Disorders (Chongqing); China International Science and Technology Cooperation base of Child development and Critical Disorders; Children’s Hospital of Chongqing Medical University, 136 Zhongshan Er Road, Yuzhong District, Chongqing, 400014 People’s Republic of China

**Keywords:** Developmental dysplasia of the hip, Closed reduction, Avascular necrosis of the femoral head

## Abstract

**Background:**

The purpose of this study was to evaluate the efficacy of closed reduction (CR) in the treatment of developmental dysplasia of the hip (DDH) and to investigate risk factors associated with CR failure and avascular necrosis (AVN) occurrence in follow-ups.

**Methods:**

The study retrospectively included 110 patients and 138 hips with DDH diagnosis that underwent closed reduction between February 2012 and November 2015 in our single tertiary medical institution. The failure rate of CR and the underlying risk factors were evaluated. Meanwhile, the incidence of AVN and the related risk factors among the successful CR cases were assessed.

**Results:**

The overall failure rate of DDH treated by CR in the present study was 31.16% (43/138). Risk factors for the CR failure were older age at the time of CR (≥ 18.35 month), large medical interval before CR (≥ 35.35 mm), and severer dislocation of the affected hip (IDHI grades III and IV). The incidence of AVN was 8.33% (6/72) in patients with successful CR at the last follow-up. No significant risk factors had been established in the present study that associated with the AVN occurrence.

**Conclusions:**

For the treatment of DDH with CR, patients with younger age might achieve better outcomes; early diagnosis and early treatment might be the key point in the DDH treatment.

## Background

Developmental dysplasia of the hip (DDH) is a common hip deformity among infants which affects 1 to 3% of newborns [1]. The literatures suggested that the pathogenesis of DDH might be related to genetic abnormalities [2, 3]. The DDH encompasses a spectrum of disorders according to the relationship between the acetabulum and the femoral head which is ranged from mild acetabular dysplasia to hip subluxation and eventually dislocation. It has been reported that DDH is the most common cause of hip arthritis in women younger than 40 years and accounts for 5 to 10% of all total hip replacements in the USA [4]. Different treatment modalities for DDH have been well established, and appropriate procedures should be applied depending on the patient’s age and the severity of the disorder [5]. In any circumstances, the primary goal of the treatment is to achieve a stable, concentric reduction to enable normal femoral head development and continued acetabular growth and remodeling [6]. Early diagnosis and treatment for the DDH are essential to avoid further surgical interventions in some cases. A successful initial treatment of DDH with the Pavlik harness appears to restore the natural development of the hip to normal [7]. Unfortunately, many patients, especially those in developing countries, miss this early treatment window [8].

Closed reduction (CR) followed by 3–4 months of immobilization in spica casting is considered the standard method for children presenting at 6–18 months of age, whereas the success rates varied in the literature [9]. In order to promote the success of CR for the treatment of DDH, it is necessary to identify true predictors of failure [10]. In addition, the avascular necrosis (AVN) of the femoral head is the most feared and frequent complication after CR procedure. The probable etiologies and the risk factors associated to the AVN has been widely discussed, but controversies still remained, indicating a need for more rigorous identification of AVN risk factors for prognostic and preventive purposes.

Therefore, this study aims to evaluate the efficacy of CR in treating patients with DDH and to determine the risk factors for CR failure and investigate AVN occurrence among patients after preliminary successful CR.

## Methods

### Patient selection

This is a retrospective observational cohort study. After the approval from the institutional review board of the Children’s Hospital of Chongqing Medical University (No.2017001), we retrospectively screened patients who underwent CR due to DDH between February 2012 and November 2015 in our single tertiary medical institution. Our inclusion criteria were (1) patients with late-presenting DDH of more than 6 months at diagnosis and patients who failed to the prior treatment including Pavlik harness or Ilfeld abduction orthoses, (2) DDH patients with hip subluxation and dislocation (IHDI ≥ grade II), (3) patients who received CR following the bilateral long leg hip spica cast immobilization, and (4) patients and their radiographic data who were followed for at least 24 months. Exclusion criteria were (1) patients with acetabular dysplasia only or slight subluxation, (2) hip dislocation associated with a syndrome or other congenital hip abnormality, (3) patients with history of any open reduction procedure before initial CR, and (5) patients with incomplete clinical and radiographic data at presentation.

After screening, 110 DDH patients with 138 affected hips were included in the present study. There were 17 males and 93 females. There were 82 patients with unilateral DDH (82 hips) and 28 patients with bilateral DDH (56 hips). The average age at the initial treatment was 16.57 ± 4.96 months which was ranged from 6.40 to 33.20 months.

### Closed reduction procedure

**Fig. 1:**
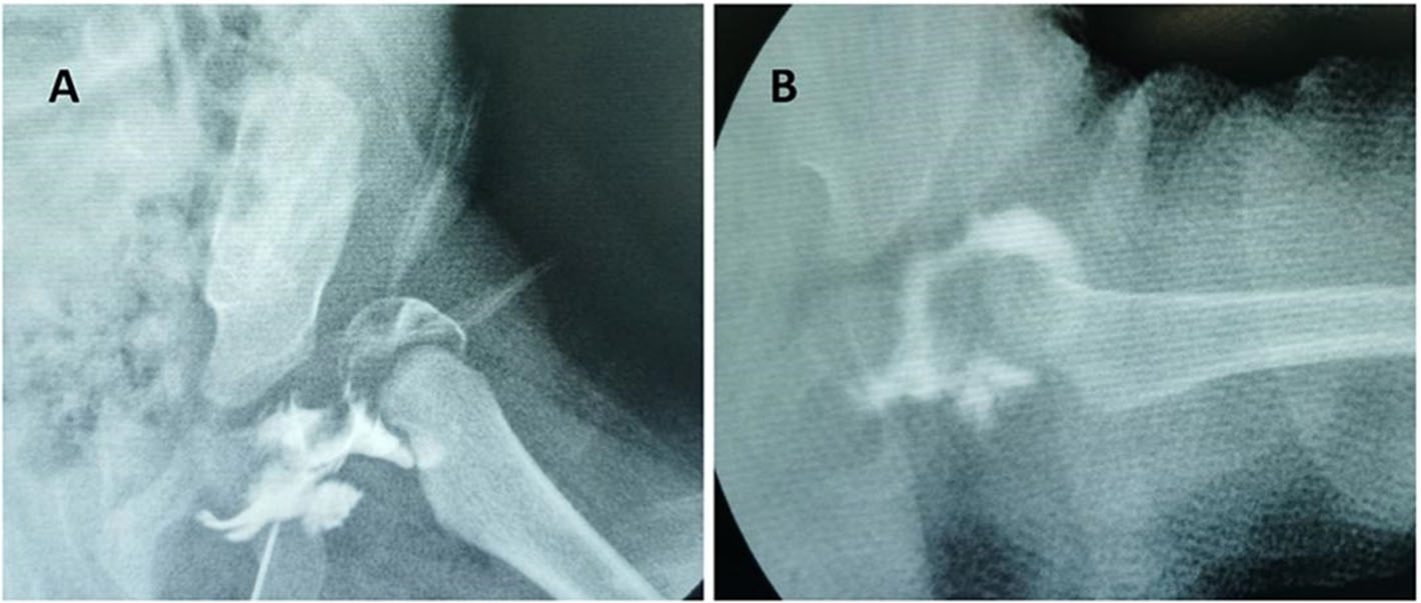
The hip was dislocated from the acetabulum before CR (**a**). The concentric reduction has been achieved after CR

Arthrography was performed in all the affected hips of included patients through an adductor longus muscle approach using 1 cm^3^ of Iohexol as a contrast to evaluate hip position and assist reduction [11]. The reduction was performed by Ortolani manoeuvre gently, and CR was considered to be achieved when the centre of the femoral head had been pulled down to a position opposite the triradiate cartilage (Fig. 1). Furthermore, if the adductor contracture impedes the hip reduction, the percutaneous adductor tenotomy was performed to reach a reliable safe zone [12]. Thereafter, as a concentric and stable reduction was achieved, the hip was immobilized by the bilateral long leg hip spica cast at 90° to 110° of flexion and 40° to 60° of abduction for 12 weeks, with a plaster change at 6 weeks. All patients were treated with an abduction orthoses after removal of the spica cast for a period of more than 3 months until concentric reduction was stable. During follow-ups, affected hips with redislocation and/or the residual acetabular dysplasia would be suggested to CR failure, and the open procedures (OR) (open reduction of the dislocated hip concomitant with innominate osteotomy and/or femoral osteotomy) would be conducted only if informed consents were obtained from these patients’ parents. All patients were followed up every 3 months in the first year after removal of the cast, and then followed up every 6 months during the second year, and every year thereafter. Anteroposterior (AP) pelvic radiographs and the frog leg lateral view were performed in all patients preoperatively at each follow-up after removal of spica cast to assess the reduction. Nevertheless, patients have only taken an AP pelvic radiograph during Spica casting immobilization. However, for a hip with an uncertain reduction, a CT scan or MRI would be further employed for intensive evaluation. All the enrolled patients’ radiographs were reviewed individually by two researchers (Y. Z. and G. Z.), and all the classifications were determined by two authors with a consensus.

### Radiographic evaluation before initial closed reduction

#### IHDI (International Hip Dysplasia Institute classification)

The degree of the hip dislocation was assessed on the basis of the IHDI classification [13].

#### Presence of ossific nucleus of the femoral head

The presence of a proximal femoral ossific nucleus in each patient was reviewed and recorded based on the pelvic plain radiographs before the initial CR.

#### AI (Acetabular Index) measurements

The AI was measured on the AP pelvic radiographs to evaluate the acetabulum developmental situation at the time of CR [14].

#### MI (Medial interval) after CR

The medial interval was defined as the vertical distance from the medial edge of the ischium to the middle point of the proximal metaphyseal border of the femur [15].

#### Osteonecrosis of the femoral head

The definition of femoral head osteonecrosis was graded according to the Bucholz-Ogden system [16]. The Bucholz-Ogden types I and II were not currently thought to affect the functional and radiographic outcomes at skeletal maturity [17]. We therefore defined types III and IV as the femoral head osteonecrosis in the present study.

#### Severin classification

The radiographic outcomes were assessed on the basis of the Severin radiographic classification [18]. Severin types I and II were considered a success of CR; however, types III, IV, V, and VI were considered a failure of CR.

### Primary outcomes

Our primary outcomes were to evaluate the efficacy of CR in the treatment of DDH and to further investigate the underlying risk factors associated to the CR failure. Failure of CR was defined as follows: (1) a hip that underwent OR procedures (open reduction of the hip with/without osteotomies) owing to the redislocation or persistent acetabular dysplasia after initial CR and (2) a hip with a grade range from III to VI according to the Severin radiographic classification at the latest follow-up. For the determination of the risk factors related to the CR failure, it is logical to adopt cases instead of hips as the independent variable because the demographic data (age, sex, etc.) were unique in each case with bilateral DDH. Therefore, cases would be defined as failure even if only a single side failure occurred in the bilateral DDH.

### Secondary outcomes

Osteonecrosis of the femoral head after CR in the treatment of DDH was also a widely concerned issue. Therefore, we further assessed the AVN occurrence among the cases with preliminary successful CR.

### Statistical analysis

All variables were analyzed by SPSS 22.0. Statistical software, and continuous data were indicated by X ± SD. Chi-square test and ANOVA analysis were used for univariate comparison, and binary logistic regression analysis was used for multivariate analysis. In investigating the relevant risk factors, the ROC curve was used to determine the grouping node, and the AUC > 0.5 was considered as the model having predictive value. The level of statistical significance was determined with the *P* value set at 0.05 (*P* ≤ 0.05).

## Results

After screening, there were 110 patients with 138 hips included in present study. Patient demographics and radiographic findings are shown in Table 1.

**Table 1:** Patient demographics of 110 patients included in the study

Number of patients/hips (*n*)	110 patients/138 hips
Age at initial CR (Mo)	16.57 ± 4.96 (6.40 to 33.20)
Follow-up (Mo)	51.22 ± 13.35 (24.03 to 79.37)
AI at the initial CR (°)	36.48 ± 6.17
MI after CR	36.28 ± 6.16
Sex (*n*)	
Male	17 patients
Female	93 patients
Laterality (*n*)	
Unilateral DDH	82 patients
Bilateral DDH	28 patients
Percutaneous adductor tenotomy (*n*)	
Yes	64 patients/85 hips
No	46 patients/53 hips
Presence of femoral ossific nucleus (*n*)	
Yes	64 patients/120 hips
No	15 patents/18 hips
IHDI grade (*n*)	
II	29 patients/37 hips
III	40 patients/48 hips
IV	41 patients/53 hips

As described in the “Methods” section, the failure of CR treatment was as defined hips which underwent OR procedures following CR treatment or hips with Severin grade III or above at the latest follow-up. There were 27 patients with 32 hips underwent OR following CR treatment owing to the recurrence of dislocation (10 hips) or sustained acetabular dysplasia (22 hips) at any time during follow-ups. Among the patients who underwent OR procedures, 19 hips were from 19 unilateral DDH patients and 13 hips were from 8 bilateral DDH patients. Among bilateral cases, 10 hips in 5 bilateral DDH patients underwent bilateral OR and 3 hips in 3 bilateral DDH patients underwent single side OR. And there were 11 patients with 11 hips defined as failure because of the unsatisfactory Severin grading (grade III or more), including 10 hips from 10 unilateral DDH patients and 1 hip from a bilateral DDH patient with a single side failure. In conclusion, the overall failure rate of DDH hips treated by CR in the present study was 31.16% (43/138).

**Fig. 2:**
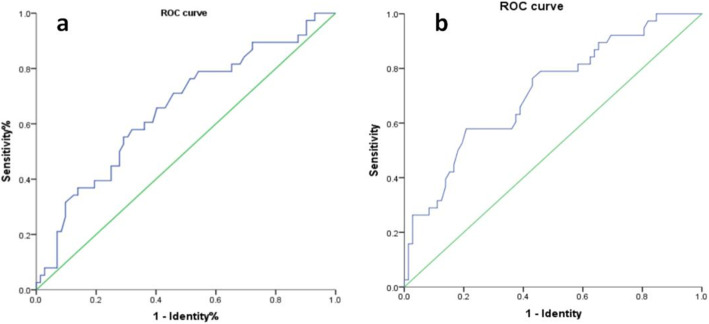
The ROC curve of the age at initial CR (**a**). The ROC curve of MI after CR immediately (**b**)

For the inclusion of the hip radiographic indices to investigate the prognostic factors of CR, we included the 82 affected hips of the 82 unilateral DDH cases and 4 affected hips of the 4 bilateral DDH cases with a single side failure occurrence into analysis. Otherwise, we selected the left hips and their radiographic indices into analysis of cases considered as bilateral success (19 patients) or failure (5 patients) among bilateral DDH cases because the general preponderance of the left hip is the frequently involved side in DDH. Ultimately, 110 patients with 110 hips were included for the prognostic factor evaluation in the CR treatment. According to the different endings, there were 72 cases in the successful group and 38 cases in the failed group. In the univariate analysis, the mean age at the CR was significantly older in the failure group than that in the satisfactory group (15.72 ± 4.74 vs. 18.17 ± 5.04, *P* = 0.013). We constructed a receiver operating characteristic (ROC) curve of the age at CR treatment and demonstrated that the optimal cutoff point was 18.35 months (area under the curve [AUC] = 0.655, 95% CI = 0.547 to 0.763, *P* = 0.008) (Fig. 2a). Cases that underwent initial CR at lower age (≤ 18.35 month) were significantly more likely to result in a satisfactory outcome than those at older age (> 18.35 month) (*P* < 0.001). The MI after CR immediately was higher in the failed group than that in the success group (*P* < 0.001). The ROC curve established the cutoff at 35.35 mm, and cases with MI less than 35.35 mm showed a significantly higher successful CR rate (*P* < 0.001) (Fig. 2b). Cases classified with IHDI grade II were significantly more likely to result in a success than those with grade III (*P* = 0.048) or grade IV (*P* = 0.002). On the contrary, there was no significant difference between the two groups among other prognostic factors (Table 2). Furthermore, the binary logistic regression model retained initial age at CR and MI after CR immediately as the significant diagnostic variable (Table 3).

**Table 2:** The univariate analysis of the risk factors related to the CR in the treatment of DDH

	Satisfactory group	Unsatisfactory group	*P*
Number of patients (*n*)	72	38	
Age at initial CR (Mo)	15.72 ± 4.74	18.17 ± 5.04	0.013
MI after CR	31.38 ± 4.75	34.69 ± 3.81	< 0.001
Age grading at initial CR (*n*)			0.007
≤ 18.35 months	48	16	
> 18.35 months	24	22	
Sex (*n*)			0.532
Male	10	7	
Female	62	31	
Laterality (*n*)			0.757
Unilateral DDH	53	29	
Bilateral DDH	19	9	
MI after CR			< 0.001
< 35.35 mm	57	16	
> 35.35 mm	15	22	
Seniority of orthopedists (*n*)			0.137
≤ 15 years	25	8	
> 15 years	47	30	
Presence of femoral ossific nucleus (*n*)			0.49
Yes	61	34	
No	11	4	
IHDI grade (*n*)			0.010
II	25	4	
III	26	14	
IV	21	20	
AI at the initial CR (°)	35.92 ± 6.49	37.53 ± 5.45	0.196

**Table 3:** The Binary logistic regression model of the risk factors related to the CR in the treatment of DDH

	Regression coefficient	95% CI of coefficient	Odds ratio	*P*
Intercept (constant)	− 1.656			
Age grading at initial CR				
> 18.35 months vs. ≤ 18.35 months	0.990	1.124 to 6.447	2.692	0.026
MI after CR				
< 35.35 mm vs > 35.35 mm	1.581	2.013 to 11.741	4.862	< 0.001

To evaluate the incidence of AVN of the femoral head after preliminary success of CR, we excluded the 38 failure cases (29 unilateral cases and 9 bilateral cases) and all of their 47 hips. There were 72 patients with 91 hips enrolled in the analysis after excluding the unsatisfactory cases with their accompanied hips. We also adopted cases instead of hips into the analysis owing to the characteristics of bilateral cases mentioned above. For the radiographic data extraction, we selected the affected hip in bilateral DDH patients with a single side AVN; Otherwise, we selected the left hip in bilateral DDH patients with AVN or without AVN in two sides. The incidence of AVN was 6/72 (8.33%) assessed from the latest radiographs. Thereafter, we assessed the risk factors associated with the development of AVN after preliminary successful CR. The univariate analysis revealed that occurrence of AVN was not affected by any prognostic factors as shown in Table 4.

**Table 4:** The univariate analysis of the risk factors related to the incidence of AVN of the femoral head after CR

	non-AVN	AVN	*P*
Number of patients (*n*)	66	6	
Age at initial CR (Mo)	15.97 ± 4.67	12.98 ± 45.04	0.141
Sex (*n*)			1
Male	9	1	
Female	57	5	
Laterality (*n*)			0.936
Unilateral DDH	48	5	
Bilateral DDH	18	1	
Percutaneous adductor tenotomy (*n*)			0.308
Yes	24	4	
No	42	2	
Seniority of orthopedists (*n*)			0.086
≤ 15 years	25	0	
> 15 years	41	6	
Presence of femoral ossific nucleus (*n*)			1
Yes	10	1	
No	56	5	
IHDI grade (*n*)			0.703
II	23	2	
III	23	3	
IV	20	1	
MI after CR	31.83 ± 3.40	26.55 ± 4.64	0.008
AI at the initial CR (°)	35.80 ± 6.60	37.33 ± 5.48	0.582

## Discussion

The successful rates of CR in the treatment of DDH were inconsistent in the literature which ranged from 43 to 92 % [19, 20]. Practically, if concentric, stable reduction of the hip cannot be achieved, an OR procedure is an alternative for DDH. For efficacy evaluation of CR in the treatment of DDH, most studies only defined early CR failure as an endpoint, in which the hip did not achieve a stable reduction and need an OR [17, 21–24]. However, we believed that the hips with unsatisfactory radiographic outcomes such as residual acetabular dysplasia or subluxation in the long-term follow-ups which did not undergo further intervention should be also taken into consideration when determining the failure of CR. To avoid the affected hips which might be progressing into degenerative hip disease, most of these cases should have received further interventions to improve the congruence between the acetabulum and the femoral head. However, the fact is that not all these patients got further treatment because their parents refused owing to the asymptomatic state till the latest follow-up [25]. Consequently, the diversity failure rates of CR in the treatment DDH might be partially dependent on the different evaluating criteria for failure. It was reported that the unsatisfactory Severin grades after DDH treatment might lead to insufficient containment of the acetabulum on the femoral head which would further lead to severe degenerative hip changes [26]. Altogether, the failures should be comprised of the OR cases following CR at early stage and cases with unsatisfactory Severin grades at the last follow-up. In conclusion, the overall failure rate of DDH after CR treatment in present study was 31.16% (43/138). Furthermore, if the prognosis and the related risk factors of failure can be predicted at the time of initial CR, the parents can be informed regarding the outcome and future managements of their children. Many factors have been reported to as the risk factors for the failed CR including an older age, high dislocation grade, or large AI [10, 27]. It has been documented that age is an important prognostic factor in the treatment of DDH with CR, and a patient over the age of 18 months at the initial CR is likely to be associated with a poor prognosis [28–31]. Herein, we observed the similar outcomes that patients older than 18.35 months at the age of CR might progress to poor outcomes when compared with younger patients. Altogether, we concluded that using CR as a treatment regime for DDH among patients whose age over than 18 months might not be a reasonable choice.

The failure rate of CR in treating DDH was increased with the severe grading of the dislocation of the hips [32]. The higher dislocation grading correlates with an increased risk of following open reduction procedures [33]. In present study, our results also showed that the more severe dislocation of the DDH before CR was significantly associated to the inferior outcomes after CR. The successful rate in IHDI grade II was significantly higher than that in grade III (*P* = 0.048) or grade IV (*P* = 0.002). There is no difference in successful rate between grade III and grade IV (*P* = 0.209), whereas the failed incidence in grade III was 35% (14/40) which was also lower than that in grade IV, 48.79% (20/41). We inferred that more included cases in future research might demonstrate more predictable outcomes. Theoretically, there are more soft tissues between the femoral head and the acetabulum in the affected hips with higher IHDI grade, and the pressure between the femoral head and the acetabulum was greater after the closed reduction that would be acting as the obstructs in the “docking” process which subsequently results in failed outcomes including redislocation, sustained subluxation, and/or insufficient acetabular remodeling [34]. Actually, in the present study, we employed the medial interval (MI) value in attempting to determine the soft tissue obstruction between the acetabulum and the femoral head after initial reduction. Our results showed that the satisfactory group demonstrated a less MI than the unsatisfactory group, and we also constructed that MI more than 35.35 mm after CR immediately might strongly indicate poor outcomes. In the present study, we included patients with a treatment history of Pavlik harness or abduction orthoses. Our results showed the orthoses treatment did not affect the CR results. However, these points should be further discussed in further studies, because the failure of orthoses treatment for DDH in infants may involve many variables, especially the compliance to the standard treatment regimen, and these patient-related variables lead to bias outcomes [35].

Avascular necrosis (AVN) of the femoral head is one of the most concerning complications following CR, which might result in hip pain, limb-length discrepancy, abnormal gait, and premature hip degenerative disease that eventually affect hip functions and need further interventions in adulthood [19]. Previous studies reported discrepant rates of AVN which were ranged from 0 to 67% [36]. Earlier studies have reported that various possible factors related to the AVN, including the age at the onset of treatment [37], genders [38], the severity of hip dislocation at treatment [39], laterality (unilateral/bilateral DDH) [40], absence of proximal femoral ossific nucleus [41], failed Pavlik harness treatment [42], or without adductor tenotomy [43]. However, either of these underlying factors was disputed [5, 44, 45]. These variations may be a consequence of natural variation due to the relatively small case numbers, different case selection, or diversities in therapeutic regimes. In the present study, our results showed that the AVN occurs in 6/72 (8.33%) of patients with satisfactory outcomes after CR. Furthermore, the occurrence of AVN was unaffected by gender, laterality, the age at CR, presence of the ossific nucleus, adductor tenotomy, seniority of orthopedists, prereduction AI, or severity of dislocation. These results were similar to the results from a recent prospective, multicenter research [45]. As AVN after CR was a multifactorial event, high-quality prospective studies with large samples are still needed to elucidate the precise risk factors associated to AVN after DDH treatment.

## Conclusion

In general, the CR is still an effective procedure for the treatment of infant and toddler patients with DDH. For DDH patients with older age and severer dislocation, it is important to keep a close watch after CR and take appropriate intervention to avoid progressive dysfunction of the hip. No determined factors had been confirmed associated with the AVN occurrence after preliminary CR success in present study.

However, there are still some limitations in present study. The AVN evaluation after CR should include more cases and longer follow-up as osteonecrosis secondary to the treatment of DDH is a relatively benign condition in children and teenagers. Errors might be introduced when radiographic induces measurements such as AI or MI, either by incorrectly positioning the child for radiographs (hip flexion/extension and rotation) or by inter- or intra-observer errors.

## Data Availability

The datasets used and/or analyzed during the current study are available from the corresponding author on reasonable request.

## Abbreviations

DDH: Develop mental dysplasia of the hip ; CR : Closed reduction ; OR : Open reduction; AI : Acetabular Index ; AVN : Avascular necrosis ; IHDI : International Hip Dysplasia Institute classification; AP : Anteroposterior ; MI: Medial interval
